# Recurrent oesophageal strictures in bullous systemic lupus erythematosus: a case report

**DOI:** 10.1093/rheumatology/keag603

**Published:** 2026-10-28

**Authors:** Foteini Moniati, John A Reynolds

**Affiliations:** Department of Inflammation and Ageing, School of Infection, Inflammation and Immunology, College of Medicine and Health, University of Birmingham, Birmingham, UK; Rheumatology Department, Sandwell and West Birmingham NHS Trust, Birmingham, UK; Department of Inflammation and Ageing, School of Infection, Inflammation and Immunology, College of Medicine and Health, University of Birmingham, Birmingham, UK; Rheumatology Department, Sandwell and West Birmingham NHS Trust, Birmingham, UK

*Rheumatology* key message

- Oesophageal stricture is a rare but clinically significant complication of bullous systemic lupus erythematosus.

Dear Editor, Bullous systemic lupus erythematosus (BSLE) represents a rare subset of systemic lupus erythematosus (SLE), affecting fewer than 1% of patients. It is characterized by acute-onset vesiculobullous eruptions with subepidermal blistering, linear deposition of immunoglobulin G and complement at the basement membrane zone (BMZ) [1, 2]. Autoantibodies to type VII collagen contribute to dermal–epidermal separation [2, 3]. We report recurrent oesophageal strictures in a patient with refractory BSLE, highlighting the therapeutic challenges of this unusual manifestation.

A 63-year-old Caucasian female had longstanding SLE, diagnosed in 1995, and antiphospholipid syndrome previously complicated by an ischaemic stroke, for which she remained on long-term warfarin. Serositis preceded her diagnosis. She subsequently developed Raynaud’s syndrome and inflammatory arthritis early in the disease course, together with oral ulcers, alopecia, livedo reticularis and mild lymphopenia. Historically, ANA and anti-dsDNA were positive with negative ENA, and C3 and C4 were intermittently low. She presented in late 2019 with oral ulceration progressing to vesiculobullous lesions involving the palms, wrists, soles and nasal vestibule. Punch biopsy demonstrated subepidermal blister with an eosinophil-rich inflammatory infiltrate, and direct immunofluorescence revealed linear IgG and C3 deposition along the BMZ. This pattern is shared with epidermolysis bullosa acquisita (EBA), bullous pemphigoid (BP) and mucous membrane pemphigoid. On salt-split skin, IgG localizes to the dermal floor in BSLE and EBA and the epidermal roof in BP, although mucous membrane pemphigoid may show either pattern [3–5]. Neither salt-split skin indirect immunofluorescence nor anti-type VII collagen serology was performed, so the diagnosis rested on the established SLE, which distinguishes BSLE from EBA, together with a blistering distribution atypical of classical BP and of the mucosa-predominant pattern of mucous membrane pemphigoid.

At the time of BSLE diagnosis, full blood count and liver function tests were unremarkable aside from neutrophilia and lymphopenia. The INR was 2.33 (target 3.5–4.5). Immunological evaluation demonstrated anti-dsDNA antibodies of 29.3 (0.0–30.0) IU/ml, with normal complement levels: C3 1.17 (0.75–1.75) g/l and C4 0.21 (0.14–0.54) g/l. There were no features of active lupus and no prior indication for systemic immunosuppression beyond hydroxychloroquine at bullous presentation.

She was commenced on prednisolone and methotrexate, alongside background hydroxychloroquine. Methotrexate was discontinued after 1 year for inadequate cutaneous control and replaced by mycophenolate mofetil (MMF), supplemented 4 months later by rituximab as blistering persisted. Mepacrine was added 5 months afterwards following dermatology multidisciplinary review. Six months later MMF was withdrawn and dapsone commenced after breakthrough tongue and throat blistering. These escalations were directed at cutaneous and oral mucosal activity, which drove immunosuppressive decisions for most of the disease course.

From 2022, she developed progressive dysphagia and odynophagia with globus sensation, increased viscid secretions and blood-streaked saliva, reducing oral intake and causing recurrent admissions. Barium swallow demonstrated a stricture at T2 (3 mm diameter, 6 mm length) and a second near the aortic arch (1.3 mm diameter, 14 mm length) (Fig. 1A, B).

**Figure 1:**
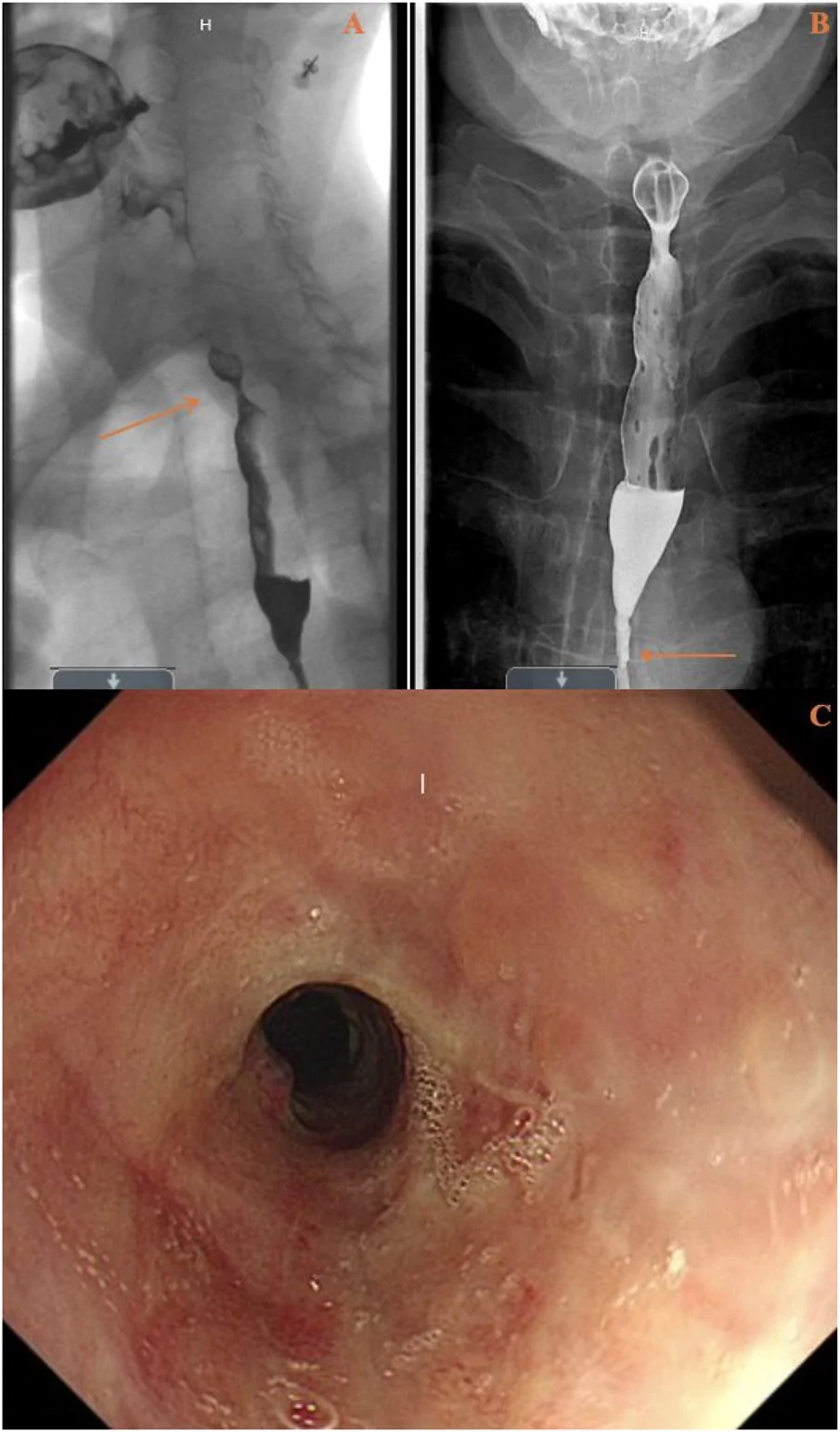
(**A**) Barium swallow (sagittal view) demonstrating a short-segment stricture at the level of T2. (**B**) Barium swallow (coronal view) demonstrating a second stricture within the medial third of the oesophagus. (**C**) Oesophagogastroduodenoscopy demonstrating mid-oesophageal luminal narrowing with inflamed mucosa

Between July 2022 and December 2024, she underwent seven oesophagogastroduodenoscopies. Initial endoscopy confirmed 2–3 mm strictures at 17 cm and 24 cm from the incisors. Graded through-the-scope balloon dilatation to 12–15 mm achieved luminal patency at each session, but structural restenosis necessitated repeated dilatations. No mass lesion or candidal plaque was described at any procedure, although histological exclusion of infective or neoplastic causes was not undertaken. During this period, she was receiving prednisolone, hydroxychloroquine, mepacrine and dapsone. Azathioprine, introduced in July 2023 for persistent oral ulceration, was discontinued the following month due to severe nausea. Intravenous methylprednisolone was given three times for oesophageal inflammatory flares. At the most recent endoscopy in December 2024, Los Angeles Grade III oesophagitis involved the mid-oesophagus (22–27 cm) (Fig. 1C), with altered blood in the stomach consistent with upper gastrointestinal bleeding.

She remains on prednisolone, hydroxychloroquine, mepacrine and dapsone, tolerating soft food and fluids but not harder textures, and has been referred to a community dietician.

Oesophageal involvement in BSLE is rarely reported. Yogarajah *et al.* described BSLE-associated oesophagitis dissecans superficialis responding to systemic corticosteroids without stricture formation [6]. Reesor *et al.* reported recurrent oesophageal stricture with granular BMZ immunoreactivity, improving on escalated MMF and prednisolone [7]. Olson *et al.* described refractory proximal oesophageal strictures with linear IgG/C3 on oesophageal biopsy but no cutaneous bullae, in mixed connective tissue disease; MMF gave only partial benefit [8]. Mucous membrane pemphigoid is a recognized cause of scarring oesophageal stricture and remains the principal differential here; the eosinophil-rich infiltrate is more typical of pemphigoid than of BSLE. In the absence of the studies above, a pemphigoid-spectrum process cannot be formally excluded, although the acral cutaneous involvement and established SLE favoured BSLE.

These reports suggest a spectrum from inflammatory mucosal disease to structural narrowing, with variable improvement following escalation of immunosuppression. Our patient progressed despite MMF and subsequent immunomodulatory therapy, requiring seven endoscopic interventions over more than 2 years. The oesophageal disease developed alongside active acral blistering and severe oral ulceration, but progressed as the cutaneous disease subsequently remitted. This suggests that oesophageal involvement, once established, becomes predominantly fibrostenotic and refractory to immunomodulation even where mucosal inflammation persists, rather than reflecting differential treatment response alone.

The coexistence of Los Angeles Grade III oesophagitis, upper gastrointestinal bleeding and multilevel stricturing broadens the recognized phenotype of BSLE-associated oesophageal involvement and, together with the limited response to immunomodulation and repeated need for endoscopic intervention, suggests a particularly treatment-resistant pattern.

Oesophageal stricture is a rare but significant complication of BSLE. Persistent dysphagia warrants prompt structural assessment, multidisciplinary management and further reporting to characterize this complication.

## Data Availability

All data are included in the published article. Further inquiries can be directed to the corresponding author.

## References

[1] Contestable JJ , Edhegard KD, Meyerle JH. Bullous systemic lupus erythematosus: a review and update to diagnosis and treatment. Am J Clin Dermatol 2014;15:517–24. 10.1007/s40257-014-0098-0 25358414

[2] Vassileva S. Bullous systemic lupus erythematosus. Clin Dermatol 2004;22:129–38. 10.1016/j.clindermatol.2003.12.020 15234014

[3] Gammon WR , Woodley DT, Dole KC, Briggaman RA. Evidence that anti-basement membrane zone antibodies in bullous eruption of systemic lupus erythematosus recognize epidermolysis bullosa acquisita autoantigen. J Invest Dermatol 1985;84:472–6. 10.1111/1523-1747.ep12272402 3889170

[4] Gammon WR , Briggaman RA. Epidermolysis bullosa acquisita and bullous systemic lupus erythematosus. Diseases of autoimmunity to type VII collagen. Dermatol Clin 1993;11:535–47. 8365038

[5] Chan LS , Ahmed AR, Anhalt GJ et al The first international consensus on mucous membrane pemphigoid: definition, diagnostic criteria, pathogenic factors, medical treatment, and prognostic indicators. Arch Dermatol 2002;138:370–9. 10.1001/archderm.138.3.370 11902988

[6] Yogarajah M , Sivasambu B, Jaffe EA. Bullous systemic lupus erythematosus associated with esophagitis dissecans superficialis. Case Rep Rheumatol 2015;2015:930683. 10.1155/2015/930683 PMC4363880 25821624

[7] Reesor E , Del Papa J, Appleton T. Bullous esophagitis with severe stricturing disease, a case study of a rare systemic lupus erythematosus manifestation. J Rheumatol 2025;52:75.

[8] Olson CG , Vora H, Kröner PT, Pang M. Esophageal stricture recalcitrant to repeated dilation in complex rheumatological disease. ACG Case Rep J 2022;9:e00940. 10.14309/crj.0000000000000940 PMC9799167 36699630

